# Seasonal Trends in Public Interest for Gastrointestinal Symptoms: A 10-Year Infodemiology Analysis Using Google Trends

**DOI:** 10.7759/cureus.83969

**Published:** 2025-05-12

**Authors:** John K Appiah, Ewurabena Plange-Kaye, Evans Donneyong

**Affiliations:** 1 Internal Medicine, Geisinger Medical Center, Wilkes Barre, USA; 2 Dentistry, Columbia University, New York, USA; 3 Internal Medicine, Tamale Teaching Hospital, Tamale, GHA

**Keywords:** gastrointestinal symptoms, google trends, infodemiology, public health surveillance, seasonality

## Abstract

Online search data have emerged as a novel tool to assess public health interest and behavior. Gastrointestinal (GI) symptoms are prevalent, yet little is known about their seasonal variation from a population behavior standpoint. This study uses Google Trends to evaluate temporal patterns in public search interest for common GI symptoms over a 10-year period in the United States. Relative search volumes (RSVs) for six GI symptoms (stomach pain, heartburn, bloating, diarrhea, constipation, and bloody stool) were extracted from Google Trends spanning January 2015 to January 2025. Data were normalized, aggregated monthly, and subjected to seasonal-trend decomposition using Loess (STL) to isolate long-term and seasonal components. Peak months were determined based on the seasonal component. Stomach pain had the highest overall search volume, with a seasonal peak in January reflecting a 15.7 percent rise. Heartburn also peaked in January with a 14 percent increase. Bloating and diarrhea showed greater seasonal fluctuation, peaking in July with increases of 41.8 percent and 21.2 percent, respectively. Constipation peaked in February with an 18.9 percent rise. Bloody stool showed the largest seasonal amplitude at 63.2 percent, though without a consistent peak month. Google Trends data reveal distinct seasonal patterns in public interest for GI symptoms, with peaks mainly in winter and summer. Infodemiology tools such as Google Trends can enhance public health surveillance and help anticipate seasonal healthcare needs.

## Introduction

Gastrointestinal (GI) symptoms such as stomach pain, heartburn, bloating, diarrhea, constipation, and bloody stool represent a significant burden of disease in both primary care and gastroenterology settings. While their underlying pathophysiology is well understood, less attention has been paid to the seasonal variation in their perceived burden at the population level.

In recent years, infodemiology, the study of health-related information available on the internet, has emerged as a valuable tool for public health surveillance [1]. Platforms such as Google Trends offer insights into real-time population-level health-seeking behaviors [2] and have been applied to monitor outbreak dynamics and seasonal illnesses [3].

This study aims to investigate the seasonal variation in public interest regarding common GI symptoms over a 10-year period using Google Trends data from the United States.

## Materials and methods

Data source

Google Trends (https://trends.google.com) provides access to anonymized, normalized data on search queries over time. Relative search volume (RSV) values range from 0 to 100, representing the proportion of a topic's searches relative to the highest point on the chart.

Selection of symptoms

Six common GI symptoms were selected for analysis based on prevalence and clinical relevance: "stomach pain," "heartburn," "bloating," "diarrhea," "constipation," and "bloody stool."

Data extraction

Monthly RSVs from January 2015 through January 2025 were collected for the United States. Search terms were entered individually to retrieve data.

Inclusion and exclusion criteria

Inclusion Criteria

Google Trends data were included if the search term represented a clearly defined GI symptom, was available for the full 10-year period, and showed consistent search volume within the United States.

Exclusion Criteria

Search terms that were ambiguous (e.g., "upset stomach"), too broad (e.g., "abdominal issue"), had insufficient data, or overlapped with non-GI contexts were excluded.

Statistical analysis

The extracted data were aggregated monthly. Seasonal-trend decomposition using Loess (STL) was employed to separate each time series into trend, seasonal, and residual components. Peak months were identified by analyzing the average seasonal component for each symptom. Analyses were performed using R statistical software with the "stats" and "forecast" packages (R Foundation for Statistical Computing, Vienna, Austria).

## Results

General trends

**Figure 1:**
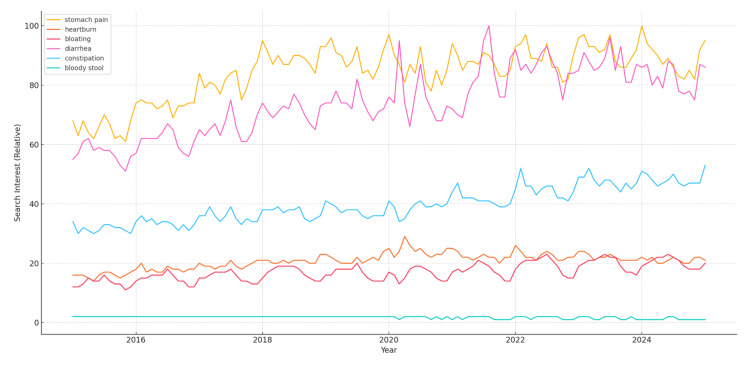
Seasonal trends in Google search interest for gastrointestinal symptoms (2015–2025) Line graphs representing relative search volume (0–100) for six gastrointestinal symptoms in the United States. Each line reflects aggregated monthly search interest over a 10-year period. Seasonal peaks are visible for most symptoms, with notable increases in winter (e.g., stomach pain, heartburn) and summer (e.g., bloating, diarrhea).

Among the six GI symptoms, "stomach pain" exhibited the highest RSV throughout the 10-year period. An overall upward trend was observed for stomach pain and heartburn (Figure 1).

Seasonal patterns

To evaluate potential seasonal trends in public interest, we analyzed RSVs for each gastrointestinal symptom over the 10-year study period. Notable seasonal variations were observed for several symptoms, suggesting potential influences of environmental, behavioral, or dietary factors throughout the year. The following outlines the observed seasonal patterns for each symptom:

Stomach pain: a consistent seasonal peak in search interest was observed each January, suggesting a recurring annual trend.

Heartburn: seasonal peaks in search volume were most prominent in January, with smaller secondary peaks observed in late November to early December.

Bloating: public interest in bloating tended to peak in July, indicating a potential summer-related influence.

Diarrhea: the highest RSVs for diarrhea occurred in July, possibly reflecting an increase in summer-related illnesses or foodborne infections.

Constipation: search interest in constipation peaked in February, coinciding with the winter months.

Bloody stool: this symptom showed consistently low search volumes throughout the study period with no discernible seasonal pattern.

The peak months for each symptom are summarized in Table 1.

**Table 1:** Peak search months for common gastrointestinal symptoms This table summarizes the peak month of relative search volume for each of the six gastrointestinal symptoms analyzed in Google Trends data from 2015 to 2025. “None” indicates no discernible seasonal peak.

Symptom	Peak Month
Stomach pain	January
Heartburn	January
Bloating	July
Diarrhea	July
Constipation	February
Bloody stool	None

Temporal patterns across years 

To complement seasonal insights, average yearly RSVs for each symptom from 2015 to 2024 were analyzed (Table 2). Stomach pain demonstrated the highest and most consistent increase in search interest, rising from an average RSV of 61 in 2015 to 73 in 2024. Heartburn also showed a steady upward trajectory, increasing from 44 to 55 over the same period. These trends suggest a growing public concern or awareness of upper GI discomfort. In contrast, symptoms such as bloating, diarrhea, constipation, and bloody stool exhibited relatively stable RSVs year over year, with minor fluctuations.

**Table 2:** Average yearly RSVs for gastrointestinal symptoms (2015–2024) This table presents the average yearly RSVs (rounded to whole numbers) for six gastrointestinal symptoms based on Google Trends data in the United States. Stomach pain and heartburn show increasing search interest over time, while other symptoms remain relatively stable. RSV, relative search volume

Year	Stomach Pain	Heartburn	Bloating	Diarrhea	Constipation	Bloody Stool
2015	61	44	34	35	34	13
2016	62	45	40	45	29	11
2017	64	46	37	43	31	10
2018	65	47	36	37	32	15
2019	66	49	32	37	33	15
2020	68	50	32	37	36	14
2021	69	51	31	38	30	12
2022	70	52	39	40	33	10
2023	72	54	36	39	34	13
2024	73	55	37	38	28	12

**Figure 2:**
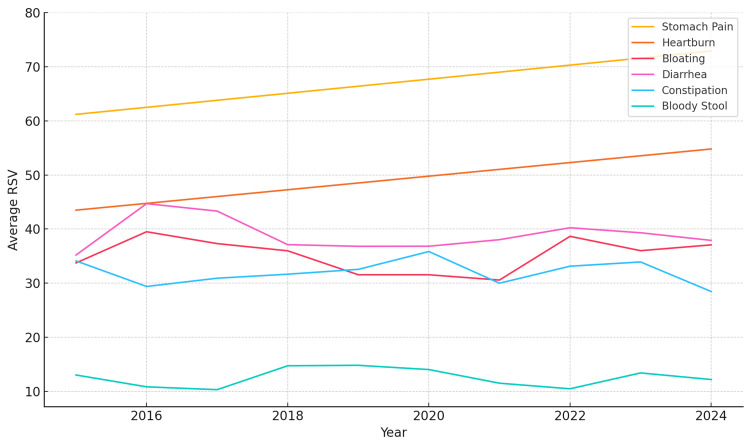
Yearly trends in average RSVs for gastrointestinal symptoms (2015–2024) Line chart showing yearly average RSVs for six gastrointestinal symptoms. Stomach pain and heartburn demonstrate upward trends, suggesting growing public interest. Other symptoms, such as bloating, diarrhea, constipation, and bloody stool, remain relatively consistent across the decade. RSV, relative search volume

These trends are visualized in Figure 2, where longitudinal patterns highlight the gradual rise in interest for stomach pain and heartburn, while other symptoms remain comparatively flat. The combination of seasonal and long-term patterns provides a more comprehensive view of public search behavior related to GI symptoms.

## Discussion

The findings of this study demonstrate that public interest in GI symptoms displays notable seasonal patterns. While stomach pain and heartburn exhibited clear winter peaks in January, their seasonal fluctuation was relatively modest at 15.7 percent and 14.0 percent, respectively. These patterns may reflect post-holiday dietary excess, alcohol consumption, and lifestyle disruptions that exacerbate upper GI symptoms [4].

In contrast, bloating and diarrhea demonstrated more pronounced seasonal variation, with July peaks corresponding to increases of 41.8 percent and 21.2 percent above their respective means. These findings may be linked to summer dietary habits, increased travel, and higher incidence of foodborne and enteric infections during warmer months [5,6]. This aligns with prior epidemiologic studies reporting elevated rates of bacterial enteritis and other GI infections during summer [6,7].

Constipation peaked in February with an 18.9 percent seasonal rise, likely due to decreased physical activity, winter-related dietary shifts, and reduced hydration [8]. Bloody stool, while showing the largest seasonal amplitude at 63.2 percent, lacked a consistent peak month. This suggests episodic public concern rather than a predictable seasonal trend, potentially triggered by media coverage or isolated awareness events rather than underlying seasonal symptomatology.

Implications for public health

Understanding these seasonal trends can guide resource allocation, targeted public health messaging, and preventive strategies. Digital surveillance tools such as Google Trends offer cost-effective, real-time insights [9,10] and have been applied to monitor infectious diseases such as dengue and COVID-19 [10].

Limitations

This study has several limitations. Google Trends data reflect search interest rather than actual disease incidence. Public interest may be influenced by media coverage, awareness campaigns, or unrelated factors. Additionally, RSVs are relative and do not represent absolute search counts.

## Conclusions

Google Trends analysis reveals distinct seasonal variations in public interest for common GI symptoms in the United States. These findings suggest that infodemiology can complement traditional surveillance methods and enhance preparedness for seasonal healthcare demands. Further studies integrating clinical data are warranted to validate and expand upon these findings.
